# Food components and contaminants as (anti)androgenic molecules

**DOI:** 10.1186/s12263-017-0555-5

**Published:** 2017-02-16

**Authors:** Daniele Marcoccia, Marco Pellegrini, Marco Fiocchetti, Stefano Lorenzetti, Maria Marino

**Affiliations:** 1 Dpt. of Food Safety and Veterinary Public Health, Food and Veterinary Toxicology Unit, Istituto Superiore di Sanità – ISS, Viale Regina Elena 299, I-00161 Rome, Italy; 2 Department of Science, University Roma Tre, Viale G. Marconi 446, I-00146 Rome, Italy; 3 Present address: Istituto Zooprofilattico Sperimentale della Lombardia e dell’Emilia-Romagna, via A. Bianchi 9, 25124 Brescia, Italy; 4 Present address: Department of Molecular Medicine, University of Padova, Via Ugo Bassi, 58/b, 35131 Padova, Italy

**Keywords:** Androgen, Androgen receptor, Endocrine disruptors, Polyphenols, Pesticides, Plasticizers

## Abstract

Androgens, the main male sex steroids, are the critical factors responsible for the development of the male phenotype during embryogenesis and for the achievement of sexual maturation and puberty. In adulthood, androgens remain essential for the maintenance of male reproductive function and behavior. Androgens, acting through the androgen receptor (AR), regulate male sexual differentiation during development, sperm production beginning from puberty, and maintenance of prostate homeostasis. Several substances present in the environment, now classified as endocrine disruptors (EDCs), strongly interfere with androgen actions in reproductive and non-reproductive tissues. EDCs are a heterogeneous group of xenobiotics which include synthetic chemicals used as industrial solvents/lubricants, plasticizers, additives, agrochemicals, pharmaceutical agents, and polyphenols of plant origin. These compounds are even present in the food as components (polyphenols) or food/water contaminants (pesticides, plasticizers used as food packaging) rendering the diet as the main route of exposure to EDCs for humans. Although huge amount of literature reports the (anti)estrogenic effects of different EDCs, relatively scarce information is available on the (anti)androgenic effects of EDCs. Here, the effects and mechanism of action of phytochemicals and pesticides and plasticizers as possible modulators of AR activities will be reviewed taking into account that insight derived from principles of endocrinology are required to estimate EDC consequences on endocrine deregulation and disease.

## Background

The increased use of plant protection products, pharmaceuticals, and plastics is coupled to the continued requests of the synthesis of new chemicals including herbicides, insecticide, biocides, active drugs, and plasticizers. Unfortunately, the increased production of new chemicals is associated with their release in the environment and, mainly through the food chain, to their potentially harmful effects on human and wildlife health. One of the most unpredictable and serious consequences of this phenomenon is the potential interference with the endocrine system of these man-made chemicals (or xenobiotics) defined as endocrine disruptors (EDs) or endocrine disrupting chemicals (EDCs). The most recent worldwide accepted definition of EDC came from the World Health Organization that made an authoritative definition of an ED as “an exogenous substance or mixture that alters function(s) of the endocrine system and consequently causes adverse health effects in an intact organism, or its progeny, or (sub)populations [1].” A recent statement of The Endocrine Society proposed a simplified definition—*an ED is an exogenous chemical, or mixture of chemicals, that interferes with any aspect of hormone action* [2] devoid of the concept of adverse or harmful effect. Such definition focus on the mode of action (MoA) and imply that a chemical interference become a significant risk depending on the chemical exposure levels [2]. Indeed, potential deleterious effects of EDCs on hormone synthesis, secretion, and action may impair cellular and tissue homeostasis. The critical windows of exposure to EDCs during the developmental ages are critical to understand their long-term effects on the physio-pathological status of the adults [2].

EDCs are a heterogeneous group of xenobiotics [3–5] which include synthetic chemicals used as industrial solvents/lubricants and their by-products (e.g., polychlorinated biphenyls/PCBs, polybrominated biphenyls, dioxins), plasticizers (e.g., bisphenol A/BPA, phthalates), food additives (e.g., semicarbazide), plant protection products such as pesticides (e.g., zineb, mancozeb and glufosinate ammonium/GA) and fungicides (e.g., vinclozolin/VIN, permethrin, chlorpyrifos), cosmetics (e.g., parabens), and pharmaceutical agents (e.g., flutamide, bicalutamide, oral contraceptives). Although the main route of exposure to EDCs for both humans and animals is diet, other different contaminated sources, such as indoor and outdoor air, water and soil, or by use of personal care products and pharmaceutical drugs could enhance EDC exposure. Currently, the discussion on the endocrine MoA centers on the hormonal systems of estrogen, androgen, thyroid, and steroidogenesis—as these are the only areas where standardized tests exist. More recently, a large body of evidence highlighted the antiestrogenic or estrogen-like effects of plant bioactives (e.g., genistein, quercetin/QRC, naringenin, resveratrol), belonging to the wide classes of polyphenols, lignans, and coumestans, frequently defined as phytoestrogens [4, 6–14]. Consequently, plant bioactive molecules are now encompassed into the EDC list of chemicals that interfere with estrogen mechanisms of action. Disappointingly, very few papers addressed the effects of these substances on androgen mechanisms of action [3, 15–18].

Here, the effects and mechanisms of action of food components and food/water contaminants (mainly phytochemical, pesticides, and plasticizers used in food packaging), acting as modulators of androgen receptor (AR) activities, are reviewed.

## Mechanisms of androgen action

**Fig. 1:**
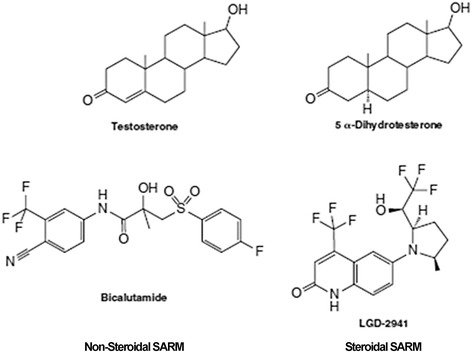
Chemical structure of testosterone, 5α-dihydrotestosterone, and two selective androgen receptor modulators (SARM)

Androgens are all steroids with 19 carbons (Fig. 1). The major naturally occurring steroids with androgenic activity are, in decreasing order of relative potency, the following: 5α-dihydrotestosterone (DHT, 150–200%), testosterone (T, 100%), androstanediol (65%), androst-4-ene3,17-dione (25%), androsterone (10%), and dehydroepiandrosterone (DHEA, 10%) [19, 20, and refs therein]. Over 95% of T is produced and secreted by Leydig cells in the testis, whereas the remaining 5% is produced in the adrenal glands by conversion of precursors (i.e., DHEA, DHEA sulfate, and androstenedione) [19]. In men, circulating levels of T range from 10 to 30 nM and decline to ≤0.3 nM after bilateral orchidectomy, whereas much lower levels (0.6–2.5 nM) are found in women. T is converted to DHT and 17β-estradiol (E2), the main active estrogen, by 5α-reductase type 1-2/SRD5A1-2 [20] and aromatase, respectively. Tissue distribution of 5α-reductase varies during the life span and the enzyme expression is hormonally regulated; for example, 5α-reductase mRNA expression in rat prostate is upregulated by DHT. The major sites of distribution of 5α-reductase in human tissues are the prostate, epididymis, seminal vesicle, and liver, while it is barely expressed in the testis, ovary, adrenal, brain, and kidney [21]. Sex hormone-binding globulin (SHBG) regulates the plasma levels and biological actions of the sex steroids; within the hypothalamic-pituitary-gonadal axis, adult Leydig cell T production depends upon the pulsatile secretion of luteinizing hormone (LH) by the pituitary gland into the peripheral circulation. LH-regulated T production and its endogenous secretion is pulsatile and diurnal with the highest peak occurring in the morning and the lowest in the evening [19].

Endogenous T levels decline in aging males, but despite the decrease in plasma T concentrations, the incidence of androgen-related pathologies, like prostate cancer (PCa) and benign prostate hyperplasia (BPH), increases with age. This increased incidence could be related to the local conversion of T to DHT being 5α-reductase upregulated [19 and refs therein].

### Androgen receptor

DHT and T bind to the same specific intracellular receptor, the AR, although DHT has two to five times higher binding affinity for AR and 10-fold higher potency of inducing AR functions than T (DHT K_d_ = 2 nM, T K_d_ = 8 nM) [21–23]. The AR, a ligand-activated transcription factor, belongs to the third group of the nuclear receptor (NR) superfamily (NR3C4, NR subfamily 3, group C, member 4) [24]. The AR is a modular protein of 919 amino acids (aa) whose structure is similar to the other NRs (Fig. 1). Four functional domains have been described in the AR: (i) an *N*-terminal domain (NTD or A/B domain, aa 1–558), with properties of transcriptional activation function (AF-1); (ii) a DNA binding domain (DBD or C domain, aa 558–624); (iii) a short hinge region (D domain, aa 624–676); and (iv) a *C*-terminal domain (E region, aa 676–919), which contains the ligand binding domain (LBD) and a coactivator binding surface (AF-2) (Fig. 1) [25, 26]. The four AR functional domains contribute differently to the overall transcriptional modulation of the AR-target gene [27]. Shortly, the AR NTD is a highly flexible and dynamic domain, whose length (60% of the whole protein) is variable due to the potential extension of the polyglutamine and polyglycine tracts [25–27].

The inactive AR is localized in the cytoplasm associated with a heath shock protein (HSP)90 chaperone complex; it undergoes to proteasome-mediated degradation in the absence of ligand [28, 29]. Upon ligand binding at the LBD, the AR undergoes conformational modifications that facilitate AR translocation to the nucleus where it dimerizes and binds to specific sequences present in target gene promoters (androgen responsive elements, AREs). Moreover, ligand binding facilitate AR intra- and inter-molecular interactions. In particular, the helix 12 of the LDB moves and together with helices 3 and 4 allow the recruitment of several transcriptional co-regulators along with the general transcription machinery complex and RNA polymerase II. The most recent compilation of AR-interacting proteins and AR-co-regulators reported the existence of 168 co-activators and 89 co-repressors, although the total number of identified AR-interacting proteins was higher than 300 [30] and refs therein. Most of these co-regulators are chromatin-modifying enzymes, namely histone deacetylases, which complexes with AR facilitating the transcription of target genes [31]. It has been proposed that the interaction between *N* and *C* termini of AR could prevent inappropriate co-regulator recruitment to the receptor until it is engaged with DNA. Intriguingly, the natural occurring atraric acid inhibits the transactivation of AR and androgen-mediated growth of AR-expressing human PCa cell lines by inhibiting the AR *N*/*C*-terminal interaction [32].

In addition to this canonical nuclear (or genomic) mechanism of action, AR-dependent, rapid (seconds to minutes) extra-nuclear mechanisms occur upon androgen treatment. These extra-nuclear mechanisms start at the plasma membrane and involve extracellular signal-regulated kinase (ERK), the phosphatidyl-inositol 3-kinase (PI3K)/Akt pathway, G protein coupled receptors (GPCRs), intracellular Ca^2+^ concentration, and cyclic adenosine monophosphate (cAMP) levels [33–37]. These data point to the existence of a membrane-bound AR. The sequence comparison between AR and the estrogen receptor identified a similar sequence for palmitoylation in both receptors [38] that was successively characterized [39]. Palmitoylation allows AR localization at the plasma membrane and its interaction with caveolin-1 (Cav-1). Cav-1 enhances AR transcriptional activity upon androgen binding to the receptor since it may increase nuclear translocation and phosphorylation of the AR [40]. On the other hand, androgen binding to AR further increases its affinity for Cav-1 [40].

As a whole, the pleiotropic effects elicited by androgens are obtained by different signal transduction pathways (i.e., nuclear and extra-nuclear), whose activation depends on the cellular context of the target cell, the AR intracellular localization (i.e., membrane-bound, cytosolic, nuclear), and the ligand itself (i.e., T vs DHT) [22].

### Physio-pathological effects of androgens

The male reproductive system comprises the paired units consisting of the testis, epididymis and vas deferens, and the penis and scrotum. The prostate, seminal vesicles, and bulbourethral glands are the male reproductive system accessory glands. The male testis have the dual responsibilities for the production and release of the germ cells and for the biosynthesis and secretion of T. Prostate plays an essential role in male reproduction secreting the prostatic fluid (highly responsive to androgens), an essential component of the seminal fluid [41, 42]. The prostatic fluid secreted by the prostate epithelium contains proteinases of the kallikrein family (e.g., prostate-specific antigen or kallikrein 3, PSA/KLK3), trace elements (e.g., zinc ions), and other molecules (e.g., citrate), all essential for the functionality of the prostate and for the subsequent activation of sperm motility [41].

Androgens are critical for male sexual differentiation, pubertal development, spermatogenesis, and maintenance of adult secondary sexual characteristics. However, androgens are pleiotropic hormones since they exert biological effects in many different non-reproductive tissues and cell types. Androgens act on the male reproductive tract inducing in utero differentiation and growth of the epididymis, seminal vesicles, and vas deferens. Prostate cell growth, function, and homeostasis are regulated by complex systemic and local mechanisms involving either the action of androgens and growth factors produced by the pituitary or the prostatic stroma [43]. After the development of the prostate gland, androgens continue to promote survival of the secretory epithelial cells, the primary cell type involved in the malignant transformation to prostate adenocarcinoma [44]. In male pubertal changes, androgens are involved in voice deepening through enlargement of the larynx and thickening of the vocal cords; moreover, they induce hair growth and distribution. Androgens exert anabolic actions on the bone tissue and skeletal muscle and modulate subcutaneous fat distribution. Moreover, they act also in the central nervous system inducing differentiation of the selected regions as hypothalamus, preoptic area, and brain cortex, and are involved in development of libido [22].

Intriguingly, the adult human male produces approximately 45 μg per day of E2, the most active within estrogens, mostly from the aromatization of T in the adipose tissue, brain, bone, breast, blood vessels, liver, and both Sertoli and Leydig cells of the testes. The T aromatization is a critical step for the closure of epiphyseal plate of the bone during puberty, for mineral resorption of the bone, and for brain function including mood and the regulatory feedback of LH production [22]. Mechanistic evidence suggests that a proportion of male reproductive endocrine disorders, including cancer, are caused by androgen insufficiency and/or by an imbalance between estrogens and androgens during critical time windows along the life cycle (e.g., pregnancy, post-natal development, puberty). However, any defects in androgen biosynthesis, metabolism, or action during development can lead to malformations such as cryptorchidism and hypospadias, as well as testicular germ cell cancer and changes in ano-genital distance [45]. These pathologies may be related components of a single underlying condition, termed “testicular dysgenesis syndrome,” originating during fetal development. In addition, cryptorchidism is a risk factor for testicular cancer, semen quality, and fertility [46].

PCa is the most frequently diagnosed non-skin malignant tumor and the third leading cause of cancer mortality in men. It is estimated that, in Western countries, about 30% of all men will develop microscopic PCa during their lifetime. PCa consists of glandular epithelial cells from the prostate gland. The tumor usually grows slowly and remains confined to the gland for many years. During this time, the tumor produces little or no symptoms or outward signs. As the cancer advances, however, it can spread beyond the prostate into the surrounding tissues and can metastasize throughout other areas of the body, such as the bones, which is the preferential metastasis site of PCa. Androgens have long been established as playing a role in the causation of PCa [47]. Although estrogens, together with androgens, play a role in normal prostate development, estrogen exposure during fetal life can profoundly alter the developmental program of the gland, sensitizing it to hyperplasia and cancer later in life [48, 49]. Androgen ablation generally leads to a decrease of PCa in a significant number of patients; however, eventually, many patients relapse with a more aggressive and metastatic stage of PCa which is androgen-insensitive, thus known as castration-resistant prostate cancer (CRPC) [50, 51].

Although the aggressive phase of PCa is androgen-independent, prostate cancerous cells still require AR to survive and proliferate. It may appear as nonsense, but many mechanisms are thought to participate to AR aberrant signaling in PCa in the absence of circulating androgens. Indeed, several AR truncated forms have been discovered in PCa, even if many of them have been also identified in non-cancerous tissues (Fig. 1). Expression of such variants, called AR-Vs, has been shown to correlate with PCa progression and CRPC. Some AR isoforms are naturally occurring as splicing variants encoded by alternative AR transcripts derived from cryptic exons downstream of the sequence for the DBD, which presents premature stop codons. Most translated AR-Vs retain the nuclear translocation domain and the DBD, but lack the LBD being constitutively active [52–55]. So far, at least 20 variants have been identified either at the mRNA or protein level [53]. Overall, AR-Vs are strongly upregulated in hormone refractory PCa and show ligand-independent constitutive transcriptional activity, thus suggesting their involvement in PCa progression and treatment resistance. AR-Vs have the potential to act alone as homo- or hetero-dimers with the full-length AR; indeed, different AR-Vs showed a different pattern of target genes that were differently modulated in the presence or absence of the full length AR [53]. Recent data [37] indicated that the extra-nuclear AR signaling may regulate nuclear AR signaling and that they may work together to coordinate gene regulation in PCa cells.

## (Anti)androgenic action of food contaminants

A meta-analysis from 1992 (resulting from 14,947 men) indicated that there had been a decline in semen quality during a period of half a century [56]. Although the results caused controversy [57], a new meta-analysis with expansion of the data to 101 studies gave similar results [58]. Although genetic factors play important roles in causing poor semen quality in some men [59], most cases of poor semen quality have no known etiology. Smoking and particularly in utero exposure to maternal smoking have been associated with reduced sperm counts [58, 60–62]. A role of EDCs has been hypothesized, but to date, there are no clear data except for some rare cases of environmental or occupational accidents where men were exposed to toxic agents like phthalates, which caused azoospermia in workers producing or using pesticides [63] or dioxin [64], which reduced semen quality. More convincingly, exposures to several antiandrogenic pesticides and/or plasticizers have been shown to induce cryptorchidism, hypospadias, and reduced semen quality in humans and rodents and are often linked to shortened ano-genital distance (typical of females) [65]. Mechanistic evidence suggests that a proportion of these male reproductive endocrine disorders are caused by androgen insufficiency and/or by an imbalance between estrogen and androgen during critical time windows along the life cycle (e.g., when the testes and genitalia are differentiating in pre- and post-natal developmental phases and/or during puberty when the organs are maturing). Finally, the upsurge in the incidence of PCa in many countries has been attributed partly to changes in diagnostic methods, namely the introduction of prostate-specific antigen (PSA) screening, but this alone cannot explain the continuing rises. Changes in PCa incidence among migrant populations and studies of twins show that environmental factors, including diet and chemical exposures, also contribute [66, 67].

### Pesticides

**Fig. 2:**
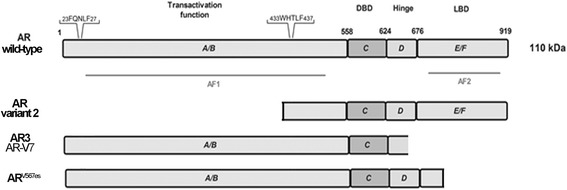
Schematic structure of wild type and variant forms of androgen receptor (AR). AR structure encompasses an A/B domain involved in protein-protein interactions via AF-1, a C domain (DBD) that engage DNA, a D domain corresponding to the hinge region, an E/F domain (LDB) containing the ligand binding domain and an AF-2 region

Pesticides are defined as substances or mixtures of substances intended for controlling, preventing, destroying, repelling, or attracting any biological organism deemed to be a pest [68]. Insecticides, herbicides, defoliants, desiccants, fungicides, nematocides, avicides, rodenticides, and hospital disinfectant (i.e., biocides) are some of the many kinds of pesticides (Fig. 2). One traditional classification of pesticides places them in one of the two groups: organic and inorganic. Organic pesticides are based on chemicals having carbon as the basis of their molecular structure, and usually do not dissolve easily in water. Inorganic pesticides are simpler compounds. They have a crystalline, salt-like appearance, are environmentally stable, and usually dissolve readily in water. Human exposure to pesticides may occur through occupational exposure in the case of agricultural workers in open fields and greenhouses, workers in the pesticide industry, and exterminators of house pests. However, exposure of the general population to pesticides occurs mainly through diet either eating food or drinking water contaminated with pesticides. Non-occupational exposure originating from pesticide residues in food, air, and drinking water generally involves low doses and is chronic (or semi-chronic) [68].

Epidemiological studies have identified pesticide application in agriculture and pesticide manufacture as associated with the PCa [69]. The exposure to six pesticides (i.e., chlorpyrifos, fonofos, coumaphos, phorate, permethrin, and butylate) out of 45 common agricultural pesticides has been correlated to increased PCa in men with a familial history. Importantly, there is a heightened sensitivity of the prostate to EDCs during puberty, thus infants and children may be considered a highly susceptible population for EDC exposures and increased risk of PCa with aging [70]. The precise mechanisms by which the chemicals related to PCa induce the carcinogenic process remain to be resolved. However, exposure to these compounds may interfere with steroid hormone metabolism in the liver and prostate altering the androgen/estrogen balance and availability that, in turn, may contribute to increased PCa risk [71]. In addition, several pesticides or their metabolites have been reported to have antiandrogenic activity via AR binding; therefore, it is not surprising that there are no reported associations between these compounds and PCa. However, this mechanism of action may cause other serious diseases. In the following sub-paragraphs, the effects of some common pesticides on androgen signaling are reported.

#### Vinclozolin (VIN) and its metabolites

VIN is a dicarboxymide fungicide, widely used on fruits and vegetables, acting as an AR antagonist in vitro and/or in vivo [72 and refs therein]. Indeed, VIN interferes with the action of androgens in developing, pubertal, and adult male rats [73, 74]. Moreover, exposure to VIN during the critical period of sexual differentiation results in sexual abnormalities expressed later in the adult male rat [75]. The mechanism evoked to explain this phenomenon is linked to the ability of VIN, as others environmental toxicants, to promote epigenetic modification [76]. Indeed, VIN exposure during fetal gonadal sex differentiation alters the epigenetic programming of the germline that can be transmitted to subsequent generations even in the absence of any exposures. This environmentally induced epigenetic transgenerational inheritance of disease is considered a component of the etiology of male infertility [76].

With the same molecular mechanism, and with almost the same potency as the classical antiandrogenic drug flutamide, the two VIN primary metabolites, M1 and M2 (Fig. 2), competitively inhibit the binding of androgens to the human AR and consequently the expression of androgen-target genes [77–79]. It has been demonstrated that VIN inhibits T-induced growth of androgen-dependent tissues (ventral prostate, seminal vesicles, and *levator ani plus* bulbocavernosus muscles) in a dose-dependent manner in the Hershberger assay using castrated immature rats treated with T [80]. In aqueous media, VIN undergoes spontaneous hydrolysis giving three metabolites called M1, M2, and M3 [81]. VIN metabolites bind to the AR [74, 82] and, acting as antiandrogens, competitively inhibit the binding of androgens to AR which leads to an inhibition of androgen-dependent gene expression in vitro and in vivo [78, 79, 83]. Recently, in an androgen-regulated human prostate cell line (LNCaP), it has been demonstrated that VIN decreases DHT-induced PSA secretion [84]. Furthermore, VIN decreased both AR nuclear accumulation and its phosphorylation in vitro [84], thus impairing the conformational changes necessary to induce the AR-mediated transcriptional activation modulated by the AF-1 region. Interestingly, the same authors have shown also a VIN effect on DHT-induced 5α-reductase (SRD5A1) gene expression in LNCaP, highlighting a further antiandrogenic effect of VIN directly on the last step of the androgen biosynthetic pathway leading to DHT formation in prostate.

#### Linuron (LIN)

LIN is a urea-derived selective herbicide used on pre- and/or post-emergence control of weeds in crops such as corn, wheat, soybeans, sorghum, cotton, carrots, and potatoes [85]. As other toxicants, LIN antiandrogenicity occurs via a dual mechanism of toxicity affecting both AR activity and T synthesis. Indeed, LIN competitively inhibits androgens binding to AR [86] and acts as a weak AR antagonist [87]. In addition, short- or long-term in utero administration of LIN did not increase the serum level of luteinizing hormone [83 and refs therein]. Consequently, it has been observed a LIN dose-dependent reduction in T production from the fetal male testis but without altered fetal Leydig cell differentiation as recognized upon in utero phthalate exposure [88].

#### Ethylene thiourea (ETU)

ETU is a common environmental contaminant, metabolite, and degradation product of the fungicide class of ethylenebisdithiocarbamateas, such as mancozeb and zineb [89]. They are used to prevent crop damage in the field and to protect harvested crops from deterioration in storage or transport [90]. Toxicological data show the thyroid gland as the primary target of ETU through the interference with thyroid peroxidase activity [91]. In addition, pre- and post-natal exposures to low doses of ETU are associated to effects on development and on the reproductive hormone profile in rats [89]. In particular, the reproductive hormone profile showed significantly reduced levels of serum DHT in male rats at ETU 0.3 mg/kg body weight/day, which corresponded to the dose at which the hypothyroid status was more evident. Severe hypothyroidism has been demonstrated to be associated with the inhibition of T conversion to DHT by 5α-reductase, with a consequent increase in serum T concentration.

#### Glufosinate ammonium (GA)

GA, the ammonium salt of the amino acid phosphinotricin, is a broad-spectrum herbicide [92] used to (i) control a wide range of weeds in agriculture, public domains, and domestic areas and (ii) to desiccate (dry off) crops before harvest. Its increased usage in several countries is derived from the approved introduction of genetically modified glufosinate-tolerant crops (such as corn, cotton, soybeans, canola, rice, sugar beets). Acute effects of GA exposure are well documented [93]. GA is a neurotoxic substance [94] and lead to neurological symptoms such as seizures, convulsions, and loss of memory [95]. Conversely, effects of long-term exposure at GA low doses remain largely unknown. In plants, GA inhibits the activity of the enzyme glutamine synthetase (GlnS) leading to a decrease of glutamine and an increase of ammonia, which entail the death of the plant [96]. In the vertebrate central nervous system, GlnS, exclusively localized in glial cells, plays a key role in the glutamate metabolism, the major excitatory brain neurotransmitter [94, 97].

Albeit GA was not reported to have any hormone-like activity, its potential influence on AR-dependent or AR-independent-mediated pathways was recently investigated by cell-based in vitro assays [98]. Interestingly, GA is not able to bind the full-length, wild-type AR as demonstrated by different in vitro gene transactivation assays including the androgen receptor AR-binding assay (ARBA), the PC-3-androgen receptor-luciferase-MMTV assay (PALM) and the AR-chemically activated luciferase expression assay (AR-CALUX) [98–101]. On the other side, in human prostate LNCaP cells, using the PSA secretion as a cell-specific, functional assay, it has been shown that GA acts as an androgen-like chemical being able to induce both free and total PSA secretion [102]. The levels of PSA secretion induced by GA at 0.01 and 0.1 mg/ml were exactly overlapping with the levels of PSA secretion induced by physiologically relevant concentration (from 2.9 × 10^−10^ and 2.9 × 10^−7^ mg/ml) of DHT. Therefore, it has been suggested that GA could act through a mutated AR bearing the point mutation T877A expressed in LNCaP cells [41].

#### Glyphosate (GLYP)

GLYP, a glycine derivate, is the active ingredient of several broad-spectrum herbicide formulations used on multiple food and non-food crops. GLYP kills plants by inhibiting 5-enolpyruvylshikimate-3-phosphate synthase, a key enzyme in the shikimate biosynthetic pathway necessary for the production of the aromatic amino acids, auxin, phytoalexins, folic acid, lignin, plastoquinones, and many other secondary products. The carcinogenic potential of GLYP, and its formulations, is a recent matter of debate at the regulatory and scientific level. Indeed, although IARC classified GLYP as a “probable human carcinogen” (IARC category 2A), due to sufficient evidence of carcinogenicity in animals, limited evidence of carcinogenicity in humans and strong evidence for two carcinogenic mechanisms have been reported and considered relevant for its toxic mechanism of action. On the other hand, EFSA reached opposite conclusions and stated that “classification and labelling for carcinogenesis is not warranted” and “glyphosate is devoid of genotoxic potential.” Such position of EFSA has been deeply criticized due to the fact that they did not gave a relevant importance to data obtained by rodent experimental models, particularly to renal carcinogenicity, as IARC did [103]. However, recent papers [104, 105] argued that glyphosate may be a key contributor to the obesity epidemic and the autism, as well as to several other diseases and conditions, such as Alzheimer’s disease, Parkinson’s disease, infertility, depression, and cancer. Indeed, these affirmations seem to be confirmed by the increased mortality of rats after 2 years of subchronic exposure to GLYP [106]. Unfortunately, the direct correlation between GLYP exposure and all these pathologies still wait for a validation. All results were hormone- and sex-dependent, and the pathological profiles were comparable. Females developed more frequently large mammary tumors than controls. Males presented up to four times more large palpable tumors, abnormal sperm morphology, and an increase of aromatase mRNA and protein levels with respect to controls [107]. This over-expression of aromatase was paralleled by the elevation of estrogen production resulting in the impairment of estrogens/androgens balance in male rats and an excess of estrogen in female rats [108, 109]. The in vitro exposure of Leydig and Sertoli cell co-cultures to the glyphosate-based formulation causes apoptosis. The exposure of drakes to GLYP resulted in alterations in the structure of the testis and epididymal region as well as in the serum levels of T and E2 [110]. All together, these data suggest that the antiandrogenicity of GLYP is mainly linked to its effect on androgen hormones metabolism that culminates in changes in the androgen/estrogen balance. However, it has been reported that GLYP exposure decreases AR expression in the testis, but do not cause detectable effects on the expression of this receptor on the efferent ductules and epididymal duct suggesting that glyphosate alone has low toxicity on male rats reproductive system [111].

### Plasticizers

**Fig. 3:**
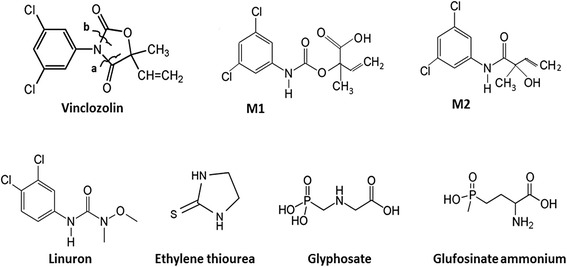
Chemical structure of pesticides commonly used in agriculture. M1 and M2 are the two primary metabolites of vinclozolin

Plasticizers are additives that increase the plasticity or viscosity of a material (Fig. 3). Plastic items containing plasticizers exhibit improved flexibility and durability. Plasticizers including di(2-ethylhexyl) phthalate (DEHP), di-isononyl phthalate (DINP), di-butyl phthalate (DBP), and bisphenol A (BPA) are commonly used in food packaging (e.g., plastic containers) and in medical devices (e.g., blood storage bags and intravenous delivery systems). In addition, BPA is a component of epoxy resins used as lacquers to coat metal products such as food cans, bottle tops, and water supply pipes. Some dental sealants and composites may also contribute to BPA exposure.

Plasticizers are not covalently bound to the polymer matrix, thus, the abrasion of the plastic/resin as well as changes of temperature and pH allow plasticizers to migrate in food; therefore, food may contain detectable levels of these compounds.

#### Phthalates

Phthalates, or phthalate esters, such as DBP and DEHP, are commonly used plasticizers, primarily necessary to soften polyvinyl chloride (PVC). High exposure levels to phthalates, in particular to DEHP, are due to their presence in medical devices [112]. Phthalate metabolites are found in the body of more than 75% of subjects sampled in the USA [113] and have been detected at median values ranging from 12.7 μg/l for benzyl butyl phthalate (BBP) to 91.8 μg/l for DBP in adult human urine samples and two to four times higher levels in the urine of children [114, 115]. Furthermore, in blood of newborn infants after transfusion, the concentrations of the DEHP were found between 3.4 and 21.6 μg/ml [116].

Although the phthalate DEHP does not bind to AR, in utero exposure to phthalates disrupts the differentiation program of androgen-dependent tissues in male rat offspring [74, 117–120].

The reproductive tract malformations in the androgen-dependent tissues are similar but not equal to the effects of antiandrogen pesticides such as VIN (see paragraph 5). Phthalates have been shown to reduce testicular T levels in fetal and neonatal male rats [121]. This decreased T production has been associated with the downregulation of genes involved in steroidogenesis [122]. The MoA of phthalates in the male involves altered Leydig cell migration and differentiation and abnormal gonocytes development [123–125]. Finally, in utero DEHP exposure altered post-natal liver development in weanling mice causing the significant and dose-dependent increase of hepatosteatosis and decreased glycogen storage [126]. At puberty, the significant decrease of glycogen storage was still present in males.

#### BPA

Considerable amounts of BPA (ranging from 0.25 to 1.11 mg/kg) have been found in randomly selected fresh food samples from an area of Southern Italy, probably deriving from plastic irrigation pipes [127]. Consequently, it is estimated that food contributes for more than 90% to the overall BPA-exposure while exposure through dust ingestion, dental surgery, and dermal absorption remain below 5% in normal situations [128]. Overall, human exposure to BPA is frequent and widespread and more than 90% of individuals have measurable amounts of BPA in urine as reported by biomonitoring studies conducted in the USA, Germany, and Canada [129 and references therein].

Exposure to BPA has been associated to a reduced proportions of male births in the populations of a number of countries, increased the risk of cryptorchidism and hypospadias, and reduced semen quality in males suggesting a possible BPA interference with the male reproductive function. However, very few data are available on BPA effects on AR transcriptional activity, while a lack of knowledge is still present on the ability of these compounds to interfere with androgen-dependent extra-nuclear signals [22, 130, 131]. BPA effects on mouse satellite cell differentiation, male rat vascular smooth muscle cells motility, and AR levels and transcriptional activity in human prostate cancer cells have been evaluated. All cell models used expressed the AR full length (i.e., 110 kDa), while prostate cancer cells were positive for several AR splicing forms (e.g., ARΔLBD or AR 75–80 kDa). Surprisingly, BPA did not impair androgen effects in normal cell lines [132, 133], but it acted as an antiandrogen in cancer cells when the AR splicing forms were expressed [132]. These data have recently been confirmed in HeLa cells transiently transfected with AR full length (110 kDa) or AR mutants (i.e., AR ~80 kDa and AR ~28 kDa) (Marino and Pellegrini, personnel communication) and have been established by other authors with different AR mutants [134]. Thus, androgen signaling seems to be less prone to BPA interference when wild-type AR is expressed, but BPA could interfere with the therapy in patients with advanced PCa via mutant ARs [134, 135].

Experiments performed in rodent models and human prostate cell lines showed that BPA can influence carcinogenesis, modulate PCa cell proliferation, and for some tumors, stimulate progression. Early life exposure to BPA may increase susceptibility to hormonal carcinogenesis in the prostate gland, possibly by developmentally reprogramming carcinogenic risk [71]. Studies using a rat model showed that brief neonatal exposure to a low dose of BPA (10 μg/kg BW/day) significantly increased the incidence and grade of prostatic intraepithelial neoplasia following adult estrogen exposure. This model of sensitivity to hormonal carcinogenesis is relevant to humans in that relative estradiol levels increase in the aging male and may contribute to prostate disease risk [136].

## (Anti)androgenic action of food components

Phytochemicals are a ubiquitous class of plant secondary metabolites; some are responsible for color and other for organoleptic properties of fruits and vegetables. A “recommended” human diet should warrant a high proportion of energy from fruits and vegetables, therefore providing, among other factors, a huge intake of phytochemicals in general considered “health promoting” by virtue of their antioxidant activity and positively modulating, either directly or indirectly, cellular and tissue redox balance [137]. However, the first cue on the antiandrogenic role of phytochemicals come from veterinary observation about sheep feed. Indeed, the adverse effect of red clover on sheep fertility in Western Australia, caused by interfering in some way with sex hormone actions, placed these substances in the class of EDCs [138]. More recently, the EDC-like role played by phytochemicals have been confirmed in in vivo experiments. Numerous effects in both male and female rats exposed to the flavonoid genistein from gestational day 7 into adulthood through placental transfer, lactational exposure, and ingestion were observed including hyperplasia of the mammary glands in both sexes and aberrant or delayed spermatogenesis [9].

### Flavonoids

**Fig. 4:**
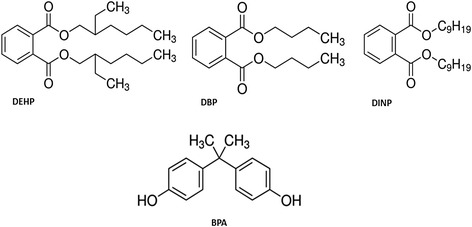
Chemical structure of some plasticizers. DEHP = di(2-ethylhexyl) phthalate, DBP = di-butyl phthalate, DINP = di-isononyl phthalate, BPA = bisphenol A

Flavonoids are widely present in fruits, vegetables, and beverages (tea, wine, beer) and in many dietary supplements and herbal remedies (Fig. 4). Quercetin (QRC) represents the most abundant dietary flavonoid found in a broad range of fruits, vegetables, and beverages, whose antioxidant and anti-inflammatory properties have been associated with the prevention and therapy of cardiovascular diseases and cancer. One of the reasons for the success of QRC (3, 30, 40, 5, 7-pentahydroxyflavone) is probably due to the relatively high bioavailability of the molecule compared to other phytochemicals. The daily intake of QRC in the diet has been estimated as 5–40 mg/day [139]. QRC, as all flavonoids, is present in food in different glycosylated forms, whereas the aglycone (i.e., the chemicals without sugar groups) is formed in phase I metabolism. Therefore, its bioavailability depends on the type of glycosides present in different food sources because it has been shown that aglycones are promptly absorbed by cells. The flavonoid glycosides are commonly hydrolyzed to their aglycones to produce effects in vivo. De-glycosylation by small intestinal epithelial cell β-glucosidases is a critical step in the absorption and metabolism of flavonoid glycosides. Flavonoid glycosides in general are absorbed as their aglycones after hydrolyzing along the digestive tract [9]. After absorption, QRC is metabolized in different organs, such as the small intestines, colon, liver, and kidney. In in vitro test, QRC appeared as mutagenic but it was not confirmed by in vivo tests in animal models, where the molecule failed to induce any significant changes when mutagenicity/genotoxicity endpoints in somatic cells were determined [140]. In 1999, IARC (the International Agency for Research on Cancer) concluded that QRC is not classifiable as carcinogenic to humans, which is in agreement with the daily intake of the molecule in the diet and the absence of known cases of adverse effects for human health [141]. QRC can be considered the prototype of a naturally occurring chemo-preventive agent due to its biological activities (antiatherogenic, anti-inflammatory, anticancer, and antihypertensive properties leading to the beneficial effects against cardiovascular diseases) [142]. Moreover, QRC caused downregulation of AR expression and activity [143] in PCa cells in which mutant ARs were expressed. AR protein expression is inhibited by QRC in a dose-dependent manner [143]. The repression effects on AR expression can actually reduce its function; moreover, QRC inhibited PSA and KLK2 secretion, two proteins known as androgen-regulated tumor markers [143, 144]. PSA and KLK2 can indirectly regulate tumor cell growth, tumor invasion, and osteoblastic metastasis [145–147]. QRC can also downregulate the expression of other prostate-specific genes, such as *NKX3.1* whose expression is associated with a more aggressive phenotype of PCa [148]. In addition, the AR-dependent upregulation of ornithine decarboxylase (*ODC*) mRNA was inhibited by QRC. The product of *ODC* gene is the key regulator of the synthesis of polyamines, which are essential for cell proliferation. *ODC* is critical in cell transformation and suggested to be a proto-oncogene [149]. It was found that ODC levels are higher in PCa compared to benign tissue [150]. QRC has an inhibitory effect on AR-regulated genes that can directly or indirectly affect cell growth. Finally, QRC can inhibit the AR expression at the transcriptional level, and thereby downregulate the androgen-inducible genes including *PSA*, *KLK2*, *NKX3.1*, and *ODC*, which play roles in development and progression of PCa. Overall, QRC has the potential to become a chemo-preventive and/or chemotherapeutic agent for PCa.

Genistein is the most abundant isoflavone in soybeans. It exhibited diverse biological activities, among these, its anticancer effects are most noteworthy [151]. Through regulating critical cell cycle genes, genistein (GEN) can inhibit cancer cell growth in vivo and in vitro. It has been reported that GEN can inhibit activation of NF-κB [152] and protein kinase B/AKT signaling pathways to induce cell apoptosis [153], both pathways are well known for their function to maintain a balance between cell survival and apoptosis. The anticancer effects of GEN have been attributed to its known inhibitory effects on tyrosine kinase, topoisomerase II, SRD5A, and angiogenesis, and its activation of several growth factor receptor pathways [154, 155]. At low, physiological concentrations, GEN binds to both the estrogen receptor subtypes (i.e., ERα and ERβ), with a greater affinity for ERβ, and GEN is thought to probably exert some or most of its effects through ER-β [156]. Moreover, GEN downregulates gene and protein expression of both AR and PSA in androgen responsive cells. However, whether GEN has a general effect on androgen responsive genes is unclear. Studies showed that there are inhibitory effects of GEN on the accumulation of products of androgen responsive genes, but the effect on mRNA levels does not always overlap, suggesting that there are different mechanisms through which GEN affects the AR signaling pathway. For example, whereas the PSA mRNA accumulation decreased in response to GEN, KLK4 mRNA levels increased. This suggests that GEN differentially affects transcriptional and post-transcriptional mechanisms in PCa. Indeed, it has been shown that GEN has a different role at both transcriptional and post-transcriptional level affecting methylation of target genes and phosphorylation of cytoplasmic proteins [157–159]. Some studies showed that GEN-treated LNCaP cells exhibit increased ubiquitination of AR, suggesting that AR protein is downregulated via a proteasome-mediated pathway. AR is normally stabilized by the chaperone activity of the heat shock protein Hsp90. The increased ubiquitination of AR after GEN treatment is attributed to decreased Hsp90 chaperone, which is more active in acetylated form. Due to the antiestrogenic activity of GEN, the histone deacetylase 6, which is an HSP90 deacetylase, is inhibited. Therefore, it is thought that AR downregulation by GEN through inhibition of the histone deacetylase 6-HSP90 co-chaperone function required stabilizing AR protein. For this, GEN could be used as a potential chemo-preventive agent for PCa along with known inhibitors of the histone deacetylase 6 and HSP90 [160].

Besides GEN, soy isoflavones consist of several types of other components, such as daidzein, the less abundant glycitein, and the metabolite equol. Daidzein is metabolized in the intestine to equol at relatively low or high levels dependent upon several biological, dietary, and presumably environmental factors. S-equol has been shown to have a modest affinity for binding to ERβ, and little affinity for ERα. Furthermore, equol (i.e., the R- and/or S-isomer) can act as an antiandrogen. Equol’s antiandrogen activity is unique as it has been demonstrated that equol does not bind AR, but specifically binds DHT with high affinity preventing the binding of AR to DHT [161]. However, there has been some controversy regarding AR regulation by soy isoflavones. Indeed, it has been reported that soy isoflavones, in particular equol, suppressed AR as well as PSA expression at the transcription level in prostate cancer cells [162]. More recently, it has been reported that equol regulates AR protein expression by activating the proteasomal pathway, thereby promoting AR degradation, without any involvement of transcriptional or translational mechanisms [163].

### Carotenoids

**Fig. 5:**
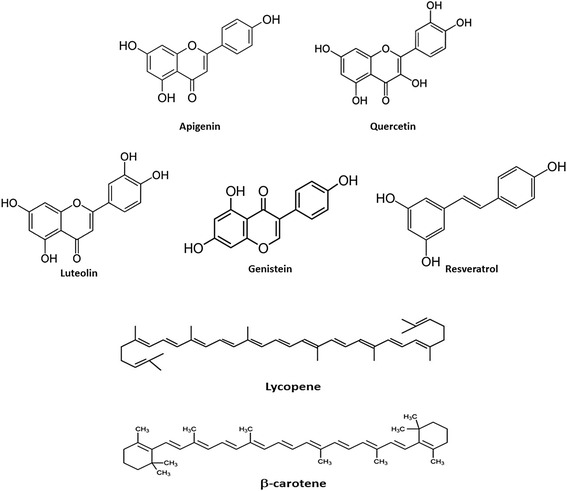
Chemical structure of some phytochemicals. Flavonoids are reported in the *first two lines* and carotenoids are reported at the *bottom*

Carotenoids are tetraterpenoid organic pigments that are naturally occurring in the chloroplasts and chromoplasts of plants and some other photosynthetic organisms like algae, some bacteria, and some types of fungi (Fig. 4). Like for other phytochemicals, animals obtain carotenoids by diets. In humans, four carotenoids (β-carotene, α-carotene, γ-carotene, and β-cryptoxanthin) have vitamin A activity and can act as antioxidants (Fig. 5) [164]. Lycopene is a bright red carotene and carotenoid pigment found in tomatoes and other red fruits and vegetables, such as red carrots, red bell peppers, watermelons, and papayas [165]. Although lycopene is chemically a carotene, it has no vitamin A activity [166]. When absorbed from the stomach, lycopene is transported in the blood by various lipoproteins and accumulates in the liver, adrenal glands, and testes. In human plasma, lycopene is an isomeric mixture containing 50% of the total lycopene as *cis* isomers. High concentration of *cis* isomers were also observed in human serum and prostate tissue [167], suggesting that tissue isomerases might be involved in in vivo isomerization of lycopene from all *trans* to *cis* form. It has been demonstrated that serum and prostate levels of lycopene in patients with PCa were significantly lower than their age-matched controls suggesting that these patients lack the ability to isomerize dietary lycopene and therefore do not absorb it efficiently [168].

## Effect of EDC mixtures

Although risk assessments have been historically conducted on a chemical-by-chemical basis, regulatory agencies are beginning to consider cumulative risk of chemicals. The effects of mixtures of chemicals like the ubiquitous phthalates and plasticizers are of concern since humans are exposed at the same time to multiple compounds [87].

Binary mixture studies were performed in rats during pregnancy exposed at dosage levels equivalent to approximately one half of the ED_50_ for hypospadias or epididymal agenesis. The binary mixtures included chemicals with different mechanism of action such as AR antagonists (i.e., VIN *plus* procymidone), phthalate esters (i.e., DBP plus BBP), a phthalate ester *plus* an AR antagonist (i.e., DBP *plus* procymidone or BBP *plus* linuron), and a phthalate ester *plus* a dioxin (DBP *plus* 2,3,7,8 TCDD). The data obtained confirmed the prediction that each chemical by itself would induce no or few malformations, but any binary mixture had led to about 50% of the males turning out to be malformed. In the same study, it has been also conducted a combinatorial mixture study exposing pregnant rats to either seven (four pesticides *plus* three phthalates) or ten (four pesticides *plus* six phthalates) different antiandrogens. The complex mixture experimental data have shown that these chemicals elicit antiandrogenic effects at two different sites in the androgen signaling pathway (i.e., AR antagonism or inhibition of the androgen synthesis). Overall, it was demonstrated that chemicals acting via disparate mechanisms display cumulative, dose-additive effects when present in combination.

In another recent study [169], conducted in vitro, 30 different AR antagonists from a wide range of sources and exposure routes (pesticides, antioxidants, parabens, UV-filters, synthetic musks, bisphenol-A, benzo(a)pyrene, perfluorooctane sulfonate, and pentabromodiphenyl ether) were tested using a gene reporter assay (MDA-kb2). Chemicals were combined at three mixture ratios, equivalent to single components’ effect concentrations that inhibit the action of DHT by 1, 10, or 20%. Concentration addition and independent action were used to calculate additive expectations. The authors have observed complete suppression of DHT effects when chemicals were combined at individual concentrations eliciting 1, 10, or 20% AR antagonistic effect. Due to the large number of mixture components, the combined AR antagonistic effects occurred at very low concentrations of individual mixture components. Therefore, a large numbers of AR antagonists from a wide variety of sources and exposure routes have the ability of acting together at the receptor to produce joint effects at very low concentrations that individually do not induce observable AR antagonistic effects.

## Conclusions

Both epidemiology studies and animal models sustain the idea that specific EDCs may influence the development or progression of male reproductive endocrine disorders including PCa [170, 171]. In large part, these effects appear to be linked to interference with estrogen signaling, either through interacting with estrogen receptors or by influencing steroid metabolism and altering estrogens/androgens balance within the body. In male, EDCs can exert prominent effects during vulnerable developmental stages as in utero or during puberty where EDCs may pose a risk of developing disease later in life. It has been theorized that the insurgence of different pathologies may be due to the exposition to EDCs during a critical window of prenatal development. Studies have confirmed that the exposure during prenatal period could alter the sex-specific characteristics and the developmental programming and could delay pubertal development without the need for a second exposure. If confirmed, these data indicate that in utero exposure to EDCs could be more critical for males which development is mainly dependent from T produced by testis in the prenatal period. Data obtained from epidemiologic evidence both in human and wildlife, in vivo studies but also genomic, proteomic, and metabolomic studies give us a picture of the effect of these compounds. However, risk assessment is usually performed on individual chemicals, but humans may be exposed to a huge number of different chemicals and chemical products from various sources and via different routes. This has raised concern about the “mixture” issue or the so-called cocktail effect. Nowadays, very few data addresses this worrying aspect of EDCs exposure. Future studies should focus on this aspect inserting phytochemicals in the mixture in order to evaluate if their protective effects against some male disease (e.g., PCa) is maintained even in the presence of food contaminants, as demonstrated for estrogen receptors and breast cancer [7].

As a whole, the combined effect of EDCs on androgen-dependent gene expression and, more general, on animal physiology is very complex because many EDCs can act as modulator of AR or estrogen receptors leading to the activation and the interaction of multiple signaling pathways, and in turn, EDCs can affect reproduction and development by more than one mechanism. Moreover, the evidence that AR mutant gain the ability to utilize some EDCs (e.g., BPA) as an agonist enlarge the effect of these substances. In spite of the huge number of studies evaluating the antiandrogenic properties of EDCs, only androgen metabolism and AR or estrogen receptors transcriptional activity have been taken into consideration, while a lack of knowledge is still present on the ability of these compounds to interfere with steroid-dependent extra-nuclear signals. Since the alteration of androgen signaling can induce a variety of endocrine disruptive responses, further studies are required to identify the downstream targets of EDC-modulated AR signaling, in order to elucidate their specific impact on male health.

## Abbreviations

AF-1: Transcriptional activation function
AR: Androgen receptor
ARBA: Androgen receptor binding assay
AR-CALUX: Androgen receptor-chemically activated luciferase expression assay
ARE: Androgen responsive element
BBP: Benzyl butyl phthalate
BPA: Bisphenol A
BPH: Benign prostate hyperplasia
cAMP: Cyclic adenosine monophosphate
Cav-1: Caveolin-1
DBD: DNA binding domain
DBP: Di-butyl phthalate
DEHP: Di(2-ethylhexyl) phthalate
DHEA: Dehydroepiandrosterone
DHT: 5α-dihydrotestosterone
DINP: Di-isononyl phthalate
E2: 17β-estradiol
ED: Endocrine disruptor
EDC: Endocrine disrupting chemical
ERK: Extracellular signal-regulated kinase
ERα: Estrogen receptor α subtype
ERβ: Estrogen receptor β subtype
ETU: Ethylene thiourea
GA: Glufosinate ammonium
GEN: Genistein
GLYP: Glyphosate
GPCR: G protein coupled receptor
HSP: Heath shock protein
KLK3: Kallikrein 3
LBD: Ligand binding domain
LH: Luteinizing hormone
LIN: Linuron
MoA: Mode of action
NR: Nuclear receptor
NTD: N-terminal domain
ODC: Ornithine decarboxylase
PALM: PC-3-androgen receptor-luciferase-MMTV assay
PCa: Prostate cancer
PCB: Polychlorinated biphenyl
PI3K: Phosphatidyl-inositol 3-kinase
PSA: Prostate-specific antigen
PVC: Polyvinyl chloride
QRC: Quercetin
T: Testosterone
VIN: Vinclozolin
